# Arrestins contribute to amyloid beta-induced cell death via modulation of autophagy and the α7nAch receptor in SH-SY5Y cells

**DOI:** 10.1038/s41598-017-01798-x

**Published:** 2017-06-13

**Authors:** Yi-qing Liu, Meng-qi Jia, Zhao-hong Xie, Xiao-fei Liu, Hui-Yang, Xiao-lei Zheng, Hui-qing Yuan, Jian-zhong Bi

**Affiliations:** 1 Department of Neural Medicine/Key Laboratory of Translational Medicine on Neurological Degenerative Disease, Second Hospital of Shandong University, Jinan, 250033 China; 2 Department of Biochemistry and Molecular Biology, School of Medicine, Shandong University, Jinan, 250012 China

## Abstract

Amyloid β-protein (Aβ) is believed to contribute to the development of Alzheimer’s disease (AD). Here we showed that Aβ_25-35_ rapidly caused activation of autophagy, subsequently leading to reduction of autophagy associated with cellular apoptosis. Further investigation revealed that the accumulation of β-arrestin 1 (ARRB1) caused by Aβ_25-35_ contributed to the induction of autophagic flux. The depletion of ARRB1 led to decreases in the expression of LC3B, Atg7, and Beclin-1, which are essential for the initiation of autophagy. ARRB1 depletion also reduced downstream ERK activity and promoted Aβ_25-35_-induced cell death. As with ARRB1, transient upregulation of ARRB2 by Aβ_25-35_ was observed after short treatment durations, whereas genetic reduction of ARRB2 caused a marked increase in the expression of the α7nAch receptor at the cell surface, which resulted in partial reversal of Aβ_25-35_-induced cell death. Although expression of both ARRB1 and ARRB2 was reduced in serum from patients with AD, the levels of ARRB1 were much lower than those of ARRB2 in AD. Thus, our findings indicate that ARRB1/2 play different roles in Aβ_25-35_ cytotoxicity, which may provide additional support for exploring the underlying molecular mechanism of AD.

## Introduction

Alzheimer’s disease (AD) is a progressive neurodegenerative disease and is the most common form of dementia, possibly contributing to 60–70% percent of worldwide dementia cases^1^. Although many efforts have been made to understand the development, pathology, and neurochemistry of AD, the mechanisms underlying this disease are still unclear^2^. Compelling evidence demonstrates that amyloid-β (Aβ) protein-induced neurotoxicity is a major pathological mechanism of AD^3^ and leads to neuronal cell death when this protein abnormally accumulates in the cortex and hippocampus in the brains of AD patients^4, 5^. Aβ is a 39- to 43-amino-acid peptide produced from the sequential cleavage of the amyloid precursor protein (APP) by β- and γ-secretases. Among these peptides, fragment Aβ_1-40_ and Aβ_1-42_, which are the two most common forms of the peptide—display more toxic effects and are prone to aggregate, contributing to the presence of extracellular amyloid plaques, intra-neuronal neurofibrillary tangles, and cerebral atrophy^6, 7^. Aβ_25-35_ is a synthetic peptide composed of 11 amino acids that corresponds to a fragment of Aβ_1-40_ and Aβ_1-42_, and is widely used for the establishment of cell models of AD^8–10^. Accumulation of Aβ results from abundant Aβ generation and reduced clearance. Intracellular Aβ has been detected in subcellular compartments such as the mitochondria, Golgi, endoplasmatic reticulum (ER), lysosomes, and cytosol, implicating sites for generation of Aβ^11, 12^. Regarding of clearance of Aβ of ubiquitin-proteasome and autophagy-lysosome are responded to degrade Aβ, and both systems are dysfunctional in AD^13, 14^. There is increasing evidence that the autophagy-lysosome system, the principal clearance machinery, plays important roles in both the production and degradation of Aβ^12, 15, 16^. For example, suppression of autophagy by deletion of the autophagy marker Beclin-1 in mice increases intra-neuronal Aβ accumulation, extracellular Aβ deposition, and neurodegeneration^17^. In contrast, autophagy is activated in AD^18^, and upregulation of autophagy results in lysosomal Aβ accumulation that is essential for oxidant-induced apoptosis in neuroblastoma cells^12, 19^. Reciprocally, it may be depending on cell context and/or pathophysiological conditions, exogenous Aβ is observed for its ability to either induces or suppresses autophagy^20–23^. Aβ impairs the activation of autophagy, and reduced autophagic clearance may counteract the accumulation of some aggregation-prone proteins, such as α-synuclein, which is toxic to neurons^23, 24^. Conversely, neurons may activate autophagy as an adaptation process when Aβ burden is below the cytotoxic level^22^. Studies have indicated that inhibition of PI3K/AKT/mTOR and activation of AMP-activated protein kinase (AMPK) contributed to Aβ-induced autophagy^25^.

Since reduction of autophagy by pharmaceutical inhibitors or genetic silencing of autophagic modulators such as Beclin-1 enhances the toxicity of Aβ in neurons, leading to an increase in apoptotic cells^17, 25^, strategies to induce autophagy have been used to explore neuronal protection. For example, some compounds, including arctigenin^26^, valproic acid^27^, carbamazepine^28^, and schisandrin B^29^, induce autophagy by modulating PI3K/AKT/mTOR and MAPK, and these compounds exert neuroprotective functions by modulating the Aβ level.

Most recently, β-arrestin1 (ARRB1) has been reported to be involved in the activation of autophagy and displays a neuroprotective role during ischemic stress^30^. As important adaptors and regulators, ARRB1 and β-arrestin 2 (ARRB2) are critical in mediating receptor desensitization and internalization as well as transduction of their own signaling pathways that are involved in numerous pathophysiological processes. It has been reported that the expression of ARRB1 is upregulated and correlates well with neuropathological severity and senile Aβ plaques in the brains of patients with sporadic AD and transgenic AD mice^31^. ARRB2 is an essential factor for the activity of the orphan G protein-coupled receptor GPR3-stimulated Aβ production via receptor-ARRB2 binding and receptor internalization. These processes are associated with γ-secretase and APP, and promote cleavage of APP to generate Aβ^32, 33^. Thus, targeting β-arrestins might provide new opportunities for AD treatment^34^.

In this study, we found that ARRB1 and ARRB2 were low in the serum of AD patients, and ARRB1 is important in Aβ_25-35_-mediated transient activation of autophagy in human neuroblastoma SH-SY5Y cells. Aβ_25-35_ increases ARRB2 expression and facilitates internalization of the α7 nicotinic acetylcholine receptor (α7nAChR), leading to enhanced Aβ_25-35_-mediated neurotoxicity.

## Results

### Activation of cytoprotective autophagy by Aβ_25-35_ delays cell death in SH-SY5Y cells

**Figure 1:**
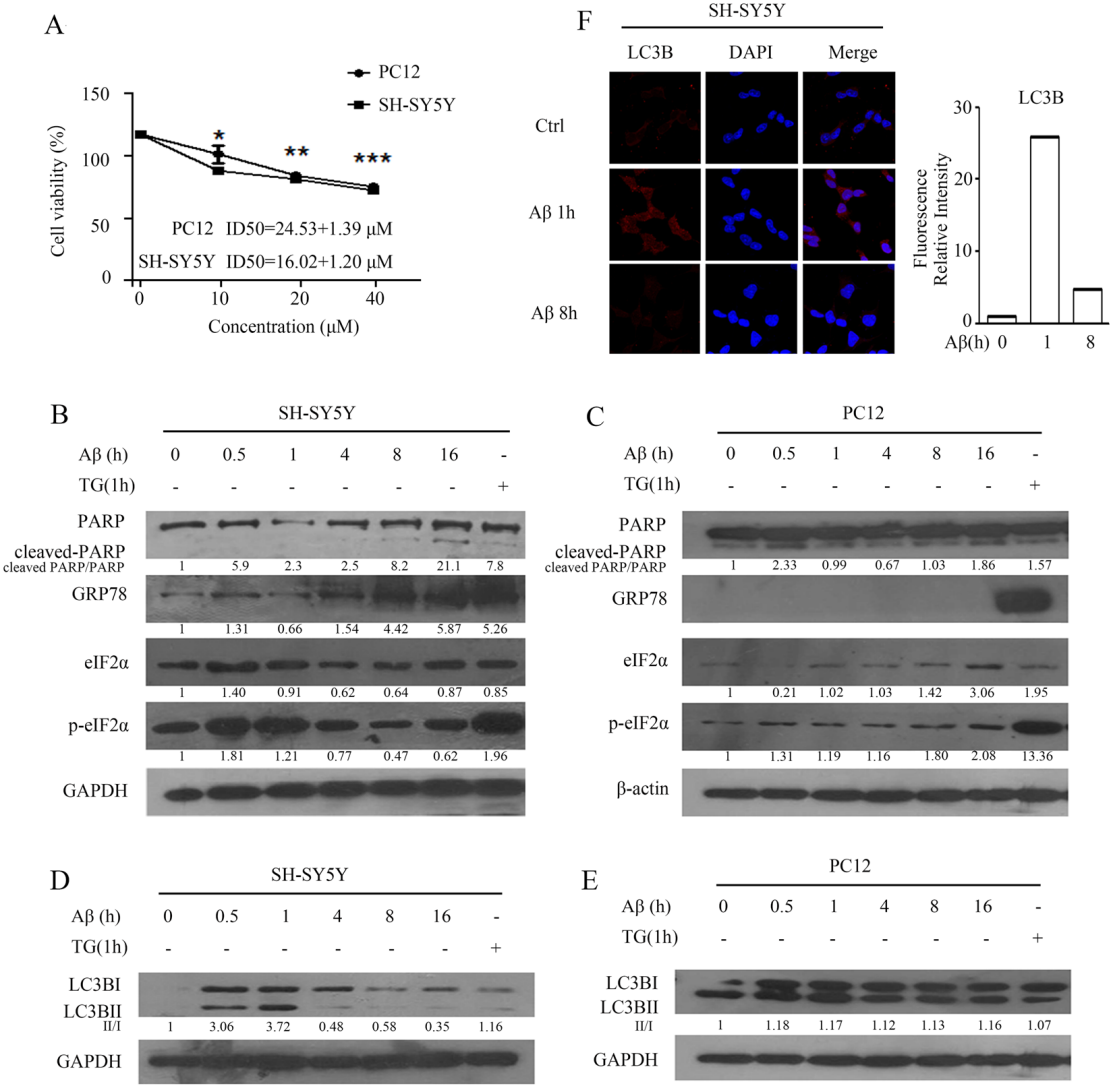
Aβ_25-35_ induces apoptosis and autophagy in SH-SY5Y cells. (**A**) Different concentrations of Aβ_25-35_ induce cell death. SH-SY5Y cells and PC12 cells. ***P < 0.001, **P < 0.01, *P < 0.05 compared with negative-control cells. (**B**,**C**) Cells were treated with 20 μM Aβ_25-35_ for 0-16 h and TG for 1 h. Markers of ER stress (GRP78, phospho- eIF_2α_) and apoptosis (PARP) were detected in cells by western blot. Figure B: SH-SY5Y cells; Figure C: PC12 cells. (**D**,**E**) Cells were treated with 20 μM Aβ_25-35_ for 0-16 h, and a marker of autophagy (LC3B) was detected in cells by western blot. Figure D: SH-SY5Y cells; Figure E: PC12 cells. (**F**) Cells were treated with 20 μM Aβ_25-35_ for 0-8 h. The red color indicates immunofluorescence staining of LC3B, showing levels of autophagy in cells. The blue color is DAPI, showing the cell nucleus. The right panel shows the merged images.The raw data of figure B/C/D/E is the figure 1B/1C/1D/1E in supplemental data.

The inhibitory effect of Aβ_25-35_ was examined in SH-SY5Y and PC12 cells, two of the most common cell lines used in AD research. Figure 1A indicates that Aβ_25-35_ exerted suppression on cell proliferation in both SH-SY5Y and PC12 cells. The IC50 value (half-maximum inhibitory concentration) of Aβ_25-35_ was 16.02 ± 1.2, and 24.53 ± 1.39 μM in SH-SY5Y and PC12 cells, respectively. This finding led us to select 20 μM Aβ_25-35_ for further mechanistic studies. Time kinetics analysis of the effect of Aβ_25-35_ on PARP cleavage, a typical apoptotic parameter, revealed that Aβ_25-35_ increased the level of cleaved PARP in SH-SY5Y cells following 8 h exposure and became evident after prolonged treatment (Fig. 1B), indicating that induction of apoptosis by Aβ_25-35_ was delayed in SH-SY5Y cells. However, cleaved PARP was rapidly induced in PC12 after 30 min treatment with Aβ_25-35_, and became weak for up to 16 h in PC12 cells (Fig. 1C). We then investigated whether Aβ_25-35_ was able to evoke an endoplasmic reticulum (ER) stress response, which is important in cell survival or cell death in response to diverse stimuli. Figure 1B shows that increased expression of glucose-related protein 78 (GRP78), a hallmark of the ER stress response, was noticeable after 30 min of exposure to Aβ_25-35_, and was maintained at a high level during prolonged treatments in SH-SY5Y cells. No detectable GRP78 was observed in Aβ_25-35_-treated PC12 cells (Fig. 1C). Thapsigargin (TG), an ER stress inducer, was used to verify the observed change in GRP78 expression resulting from ER stress. Furthermore, the phosphorylation status of the eukaryotic initiation factor (eIF_2α_) that inhibits overall protein synthesis was initially upregulated after exposure to Aβ_25-35_, and declined after approximately 4 h, at which time cleaved PARP started to appear in SH-SY5Y cells (Fig. 1B). However, phospho-eIF_2α_ levels were marginally elevated in PC12 cells during Aβ_25-35_ treatments. Thus, Aβ_25-35_ activated an ER stress response. In particular, elevated phospho-eIF2α might be involved in the delayed effect of Aβ_25-35_ on apoptosis in SH-SY5Y cells.

Given the results indicating that apoptosis was initiated after prolonged exposure to Aβ_25-35_ in SH-SY5Y cells, we reasoned that a transient protective mechanism might occur before apoptosis in response to Aβ_25-35_-induced stress. Since autophagy exerts a dual role in controlling cell survival and death, we examined level of the microtubule-associated protein 1 light chain 3B (LC3B-I) and the conversion of LC3B-I to lipidated LC3B-II (membrane-bound form), which is an autophagy marker. Figure 1D shows that levels of LC3B-I and LC3B-II were significantly increased in SH-SY5Y cells after 30 min treatment, and sharply dropped down at 1 h thereafter. There was a detectable level of basal LC3B-II in PC12 cells, which remained almost unchanged during treatment (Fig. 1E). Immunofluorescence staining further supported the observations that LC3B markedly increased after Aβ_25-35_ stimulation for 1 h in SH-SY5Y cells and was eliminated after 8 h treatment (Fig. 1F).

**Figure 2:**
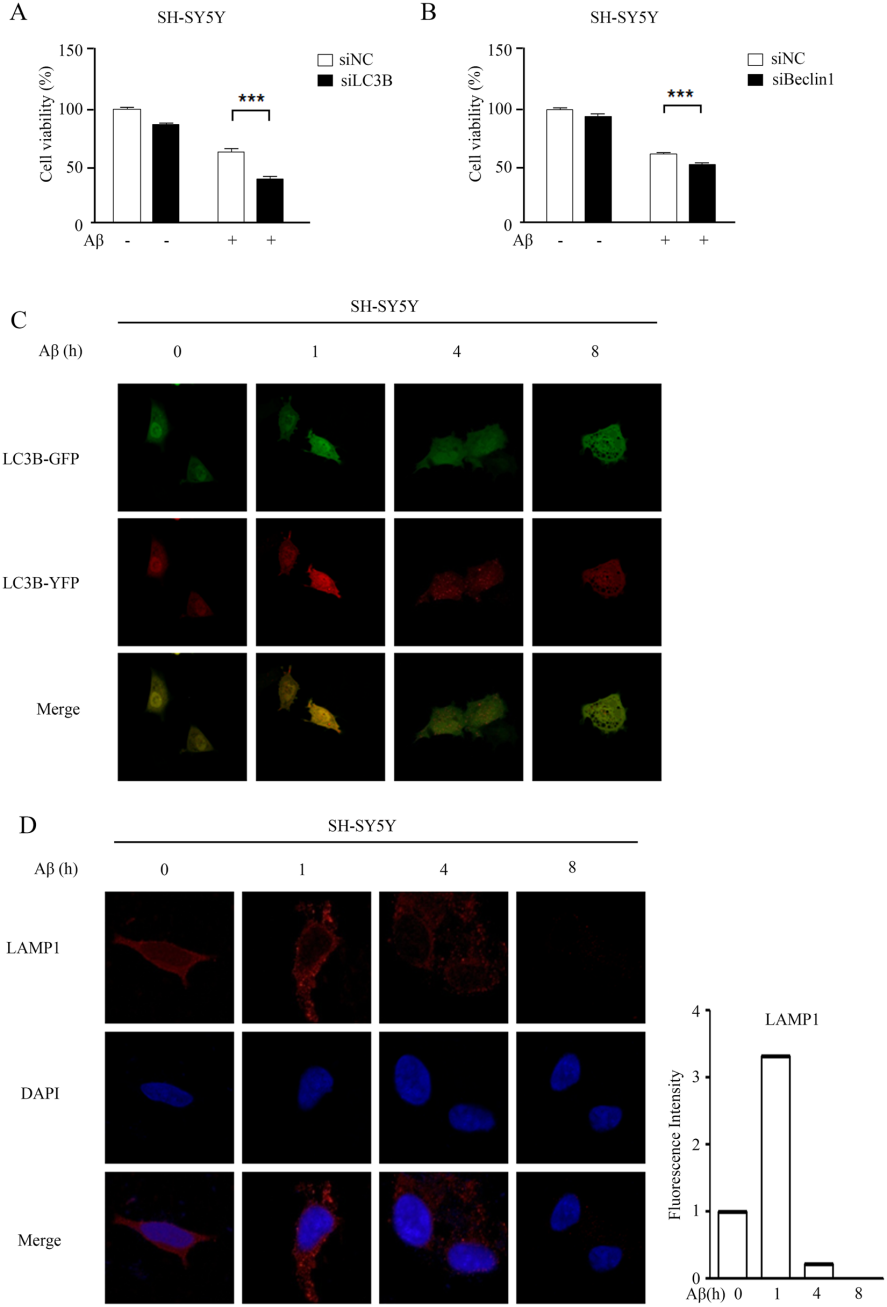
Autophagy in Aβ_25-35_-induced cell death is protective. (**A**) After knockdown of *LC3B*, the cells were treated with Aβ_25-35_ for 16 h, and cell viability was detected by MTT. ***P < 0.001 compared with negative-control siRNA- and Aβ_25-35_ -treated cells. (**B**) After *BECN-1* knockdown, cells were treated with Aβ_25-35_ for 16 h, and the cell viability was detected by MTT. ***P < 0.001 compared with negative-control siRNA- and Aβ_25-35_-treated cells. (**C**) SH-SY5Y cells were treated with Aβ_25-35_ for 0–16 h. The green color indicates LC3B on the autophagosome. The red color indicates LC3B on the autophago-lysosome, and the yellow color is the merge of the two. (**D**) Cells were treated with 20 μM Aβ_25-35_ for 0–16 h. The red color indicates immunofluorescence staining of LAMP1, a marker of the lysosome, demonstrating the number of lysosomes in cells. The blue color indicates the cell nucleus. The right panel contains the merged pictures.

To explore the role of autophagy in Aβ_25-35_-induced cell death, we genetically depleted LC3B using specific targeting siRNA in order to determine whether inactivation of autophagy would reverse the effect of Aβ_25-35_. As illustrated in Fig. 2A, knockdown of LC3B alone did not affect cell viability, whereas Aβ_25-35_-mediated cell death was elevated when LC3B was depleted in SH-SY5Y cells. Similar results were observed in cells with reduced Beclin-1, another critical regulator of autophagy (Fig. 2B). These results indicate that the activation of autophagy by Aβ_25-35_ served as a cytoprotective mechanism in SH-SY5Y cells, which may contribute to delayed apoptosis following prolonged treatments.

Since soluble Aβ-induced apoptosis is observed after prolonged treatment in primary neurons and causes lysosomal membrane damage^35^, we transfected SH-SY5Y cells with a pH-sensitive LC3-reporter plasmid containing tandem-tagged fluorescent proteins (mRFP-GFP-LC3) and analyzed the involvement of lysosomes in Aβ_25-35_-mediated apoptosis by confocal microscopy. The green LC3 puncta primarily represented autophagosomes, as GFP loses its fluorescence in acidic pH, whereas red LC3 puncta were indicative of autolysosomes. Autophagosomes appeared yellow due to overlapped red and green fluorescence in less acidic pH. Figure 2C shows that, within 1 h, both red and yellow puncta were greatly increased in response to Aβ_25-35_, indicating that increased autophagic flux occurred. However, the number of yellow puncta were markedly reduced, and puncta were mainly in red with less fluorescent intensity after 4 h and 8 h treatments in the images, indicating that disruption of lysosomes by Aβ_25-35_ led to impaired autophagosome-lysosome fusion and blocked autophagic flux. To confirm the effect of Aβ_25-35_ on lysosomal membrane damage, we examined changes in the lysosomal-associated membrane protein-1 (LAMP1) that is a lysosome marker in the presence of Aβ_25-35_. Consistent with the results shown in Fig. 2C, LAMP1-positive lysosomes were evident at 1 h, the fluorescence rapidly declined after 4~8 h treatment with Aβ_25-35_ and disappeared prior to cell death (Fig. 2D). These observations indicate that exposure of cells to Aβ_25-35_ caused the activation of autophagic flux, which facilitated early cell survival, but subsequently led to cell death, at least in part, due to the interruption of lysosomes and a failure of autophagosome maturation.

### ARRB1 contributes to Aβ_25-35_-induced autophagy in SH-SY5Y cells

**Figure 3:**
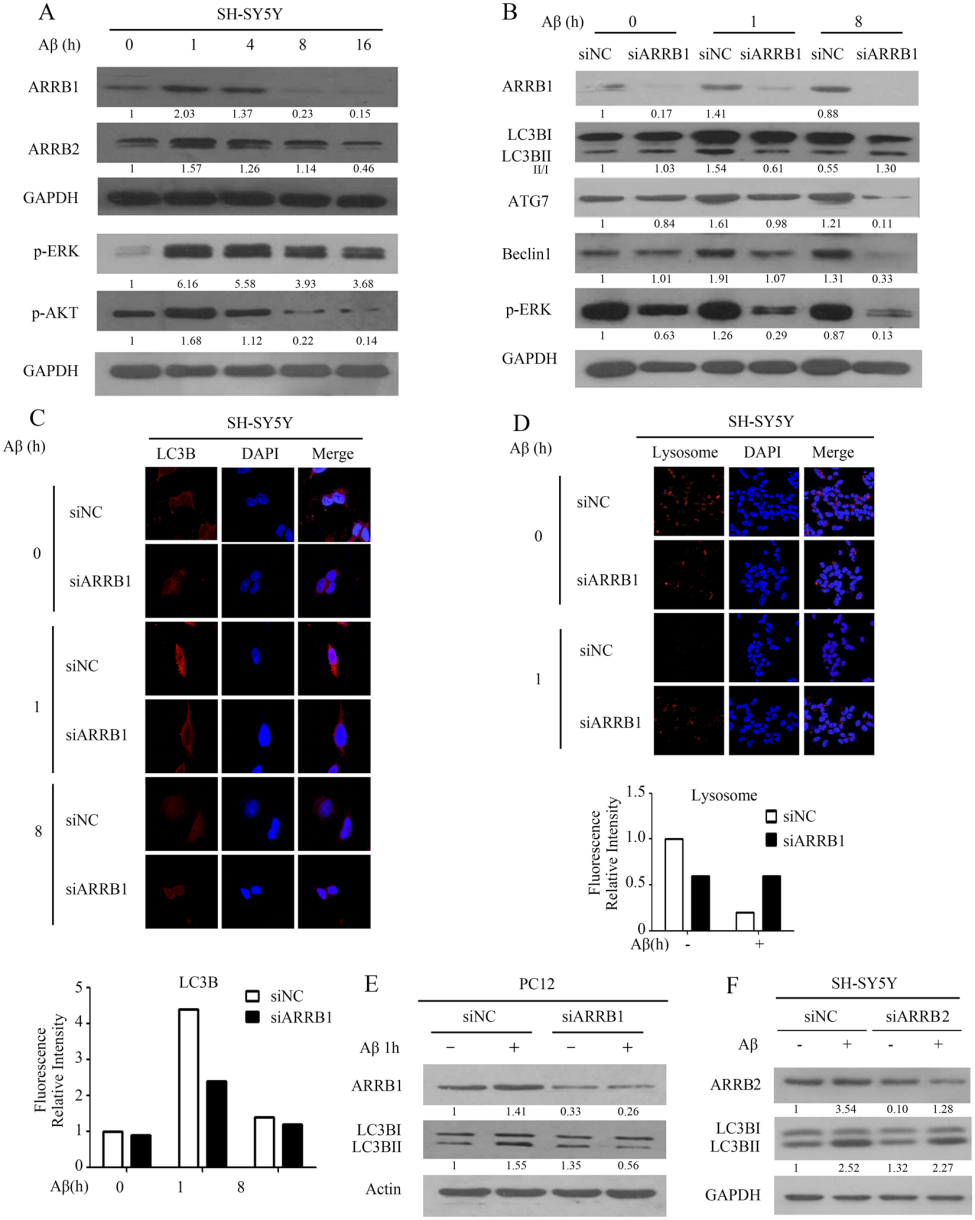
ARRB1 participates in Aβ_25-35_-induced autophagy in SH-SY5Y cells. (**A**) Cells were treated with 20 μM Aβ_25-35_ for 0–16 h, and the levels of ARRB1, ARRB2, and two survival markers (phospho-ERK and phospho-Akt) were detected by western blot. (**B**) After depletion of *arrb1*, in the presence of Aβ_25-35_, the expression of ARRB1, autophagy markers (LC3B, Atg7, and Beclin-1) and a survival marker (phospho-ERK) were detected by western blot. (**C**) With *arrb1* knockdown, in presence of Aβ_25-35_, for 1 h, the autophagy marker LC3B was detected by fluorescence staining. The red color indicates immunofluorescence staining of LC3B. The blue color is DAPI, delineating the cell nucleus. The right panel contains the merged pictures. (**D**) With *arrb1* knockdown, in the presence of Aβ_25-35_ for 1 h, the lysosome marker LAMP1 was detected by fluorescence staining. The red color indicates LAMP1 expression, and the blue color is DAPI. The right panel reveals the merge of the two images. (**E**) With *arrb1* knockdown, in the presence of Aβ_25-35_ for 1 h, LC3B was detected by western blot in PC12 cells. (**F**) With *arrb2* knockdown, in the presence of Aβ_25-35_ for 1 h, LC3B was detected by western blot. The raw data of figure A/B/E/F is the figure 3A/3B/3E/3F in supplemental data.

β-arrestins have been implicated in AD development and progression, and ARRB1 mediates neuroprotection through the activation of autophagy during ischemic stress^30^. We also found that the expression of both ARRB1 and ARRB2 was induced in response to Aβ_25-35_. Figure 3A shows that Aβ_25-35_ was able to induce the expression of ARRB1 at 1 h, which then decreased in SH-SY5Y cells. Similar to ARRB1, the levels of ARRB2 were upregulated at 1 h, and declined after prolonged treatment (Fig. 3A). In addition, the activation of ERK and Akt, two typical downstream markers for indicating the changes in the function of β-arrestins and critical for cell proliferation and survival, was observed at an early stage of treatment (1 h), and phosphorylation of these two proteins declined during the treatment period (Fig. 3A), consistent with observations in Fig. 1A that responses to Aβ_25-35_ initially stimulate cell survival and subsequently lead to cell apoptosis. Recognizing the role of ARRB1 in the activation of autophagy in cerebral ischemia^30^, we investigated whether ARRB1 was required for Aβ_25-35_-induced autophagy in neurons. As shown in Fig. 3B, the conversion of LC3BII/LC3BI was increase at 1 h exposure to Aβ_25-35_, depletion of ARRB1 resulted in a reduction of LC3B-II/LC3B-I, and decreased levels of ATG7 and Beclin-1. Autophagy was predominantly decreased at 8 h by silencing ARRB1 in the presence of Aβ_25-35_ (Fig. 3B), indicating the importance of ARRB1 in Aβ-mediated autophagy. The results in Fig. 3C further support the observations that, after exposure to Aβ_25-35_ for 1 h, LC3B fluorescence staining displayed a granular pattern in negative scramble siRNA controls, whereas in siARRB1 cells, the staining pattern was diffuse with reduced brightness. After treatment with Aβ_25-35_ for 8 h, the fluorescence intensity of LC3B was greatly decreased both in negative-control and siARRB1 cells, indicating that the *arrb1* knockdown impaired autophagy activation by Aβ_25-35_. In addition to the involvement of ARRB1 in autophagy activation, we examined the influence of ARRB1 on lysosomes in the presence of Aβ_25-35_. The results in Fig. 3D reveal that the exposure of cells to Aβ_25-35_ significantly decreased the number of lysosomes, in agreement with the results shown in Fig. 2D. However, the *arrb1* knockdown, to some extent, attenuated the inhibitory effect of Aβ_25-35_ on lysosomes, as indicated by partially restored immunofluorescence (Fig. 3D). To evaluate the relationship between ARRB1 and autophagic flux, we exposed PC12 cells to siRNA targeting ARRB1 in the presence or absence of Aβ_25-35_. Similar to the results observed in SH-SY5Y cells, abolishment of ARRB1 led to a reduced conversion of LC3B-I to LC3B-II, which was enhanced by short treatment with Aβ_25-35_ (Fig. 3E). Since the expression of ARRB2 was transiently induced by Aβ_25-35_, we also determined the role of ARRB2 in the regulation of autophagy. As shown in Fig. 3F, Aβ_25-35_ induced conversion of LC3B-I to LC3B-II, depletion of ARRB2 had limited impact on the expression of LC3BI and lipidated LC3BII, and did not affect Aβ_25-35_-actiavted autophagy. Thus, these results demonstrated the role of ARRB1, but not ARRB2, in Aβ_25-35_-activated autophagy.

### Depletion of *arrb1* enhances cytotoxicity of Aβ_25-35_ in SH-SY5Y cells

**Figure 4:**
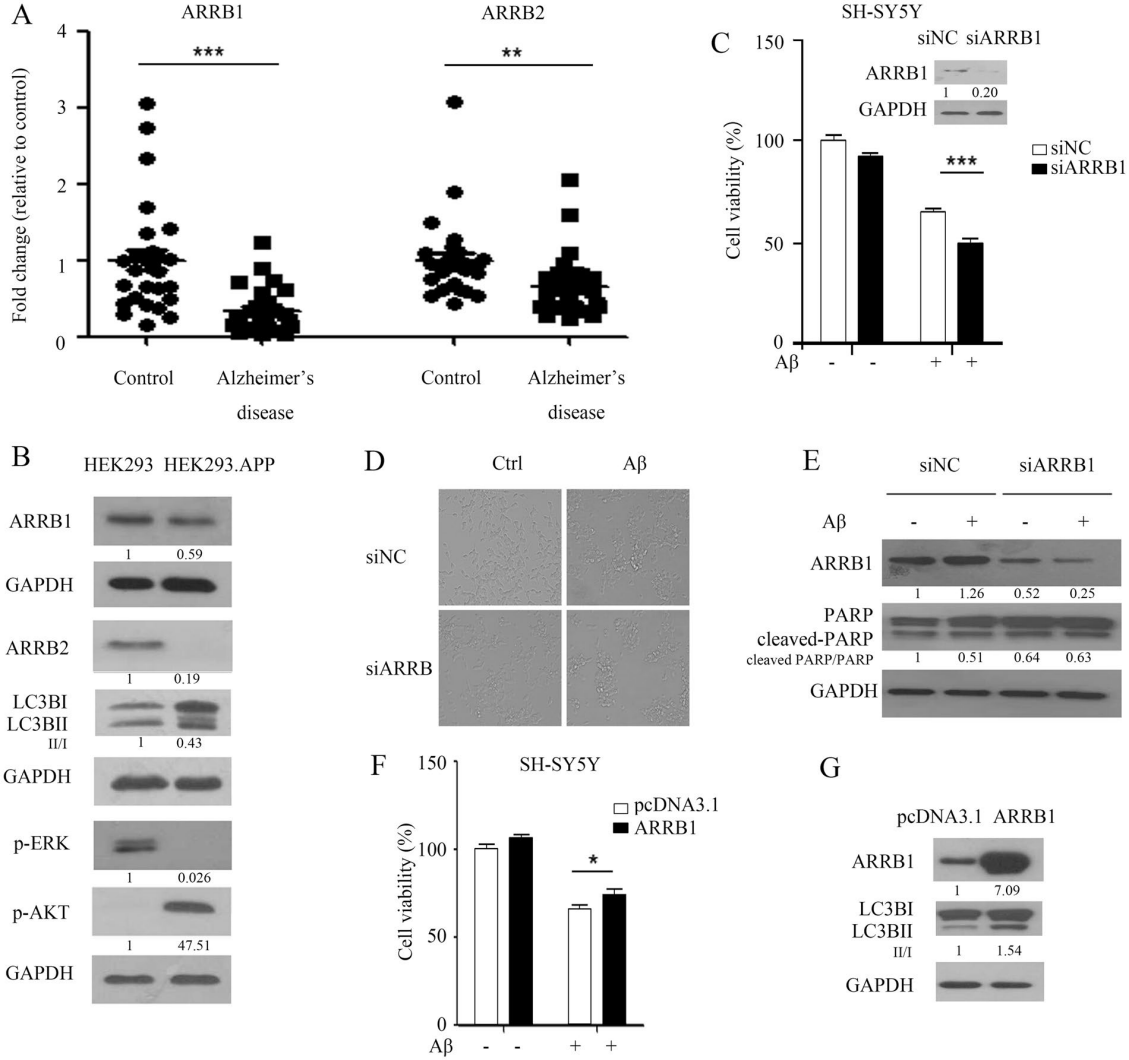
Depletion of *arrb1* enhances the cytotoxicity of Aβ_25-35_ in SH-SY5Y cells. (**A**) mRNA levels of both *arrb1* and *arrb2* were evaluated in blood samples from AD patients (N = 27) by RT-PCR. ***P < 0.001 compared to ARRB1 in healthy age-matched controls, **P < 0.01 compared to ARRB2 in healthy age-matched controls. The left panel shows mRNA level of *arrb1*, and the right panel shows mRNA level of *arrb2*. (**B**) Protein levels of ARRB1, ARRB2, a marker of autophagy (LC3B) and survival markers (phospho-ERK and phospho-Akt) were detected in cells overexpressing Aβ (HEK293^APP^) and in APPwt cells (HEK293) by western blot. (**C**) MTT of SH-SY5Y. The cells were treated with siRNA targeting *arrb1* for 48 h, and Aβ_25-35_ was added to the cells for 16 h before testing cell viability with the MTT assay. ***P < 0.001 compared with negative-control siRNA- and Aβ_25-35_-treated cells. The upper panel shows the expression of ARRB1 after knockdown of *arrb1*. (**D**) SH-SY5Y cells were treated with Aβ_25-35_ after depletion of *arrb1*, and changes in cell morphology were observed with a microscope. (**E**) SH-SY5Y cells were treated with Aβ_25-35_ after depletion of *arrb1*; cleaved PARP was detected in cells by western blot. (**F**) MTT of SH-SY5Y. Cells were treated with Aβ_25-35_ after translation of *arrb1*; Aβ_25-35_ was added to the cells for 16 h before testing cell viability with the MTT assay. *P < 0.05 compared with negative-control pcDNA3.1- and Aβ_25-35_-treated cells. (**G**) Protein levels of ARRB1 and LC3B was detected in cells by western blot. The raw data of figure B/C/E/G is the figure 4B/4C/4E/4G in supplemental data.

Since ARRB1 functions as a regulator of autophagic capacity in response to Aβ_25-35_, the role of β-arrestins in Aβ_25-35_-mediated cell death was investigated. The mRNA levels of both *arrb1* and *arrb2* were significantly reduced in human blood samples obtained from AD patients (N = 27) when compared to healthy age-matched controls (N = 27) (Fig. 4A). It appeared that the decreases in *arrb1* were more evident in AD patients compared to *arrb2* (Fig. 4A). Most AD patients develop Aβ accumulation and deposition, prompting us to test the possibility that ARBB1 and ARRB2 might also be downregulated in HEK293-APPwt cells that constitutively overexpress APP and produce Aβ. As shown in Fig. 4B, ARBB1 expression was slightly reduced in cells that overexpressed Aβ, whereas the expression of ARRB2 was lower than that of ARRB1 in APPwt cells, and was associated with a decreased conversion of LC3B-II/LC3B-I (Fig. 4B). These results indicated that persistent overexpression of Aβ led to a reduction in β-arrestin expression, which subsequently led to the inactivation of autophagy. As a downstream target of β-arrestins, phosphor-ERK was downregulated, while Akt was predominantly activated in APPwt cells, suggesting that Akt signaling may be more critical in APPwt cell survival and proliferation (Fig. 4B).

Given the role of ARRB1 in the activation of autophagy, which was decreased in AD patients, we next sought to determine whether the downregulation of ARRB1 would affect cell viability in the presence of Aβ. Transient transfection of siRNA specifically targeting *arrb1* led to a reduced amount of ARRB1 protein in cells at48 h after transfection compared to control cells transfected with nontargeting scramble siRNA, as measured by western blotting (Fig. 4C). Cell viability was reduced after silencing the expression of ARRB1, and depletion of ARRB1 facilitated Aβ-induced cell death (Fig. 4C). These results are consistent with the observation that interfering with ARRB1-activated autophagy attenuated its cytoprotective effect in response to Aβ. Parallel experiments examining changes in cell morphology again showed an increase in apoptosis of cells treated with Aβ_25-35_ after depletion of ARRB1 (Fig. 4D). Moreover, activation of cleaved PARP was observed in cells with reduced ARRB1 in response to Aβ_25-35_ compared to the group treated with Aβ_25-35_ alone (Fig. 4E). We also transiently overexpressed ARRB1 in cells to validate the protective role of ARRB1 in Aβ_25-35_-mediated cytotoxicity. Ectopic expression of ARRB1 alone had no significant effect on cell proliferation (Fig. 4F and G). However, Aβ_25-35_ induced cell death, and this cytotoxic effect was partially reversed by overexpression of ARRB1 (Fig. 4F and G). Together, these data demonstrate that interfering with the ARRB1-induced autophagic response could exacerbate Aβ_25-35_-induced cell death.

### Knockdown of ARRB2 partially protects cells from Aβ_25-35_-induced apoptosis by facilitating α7nAChR expression at the cell membrane

**Figure 5:**
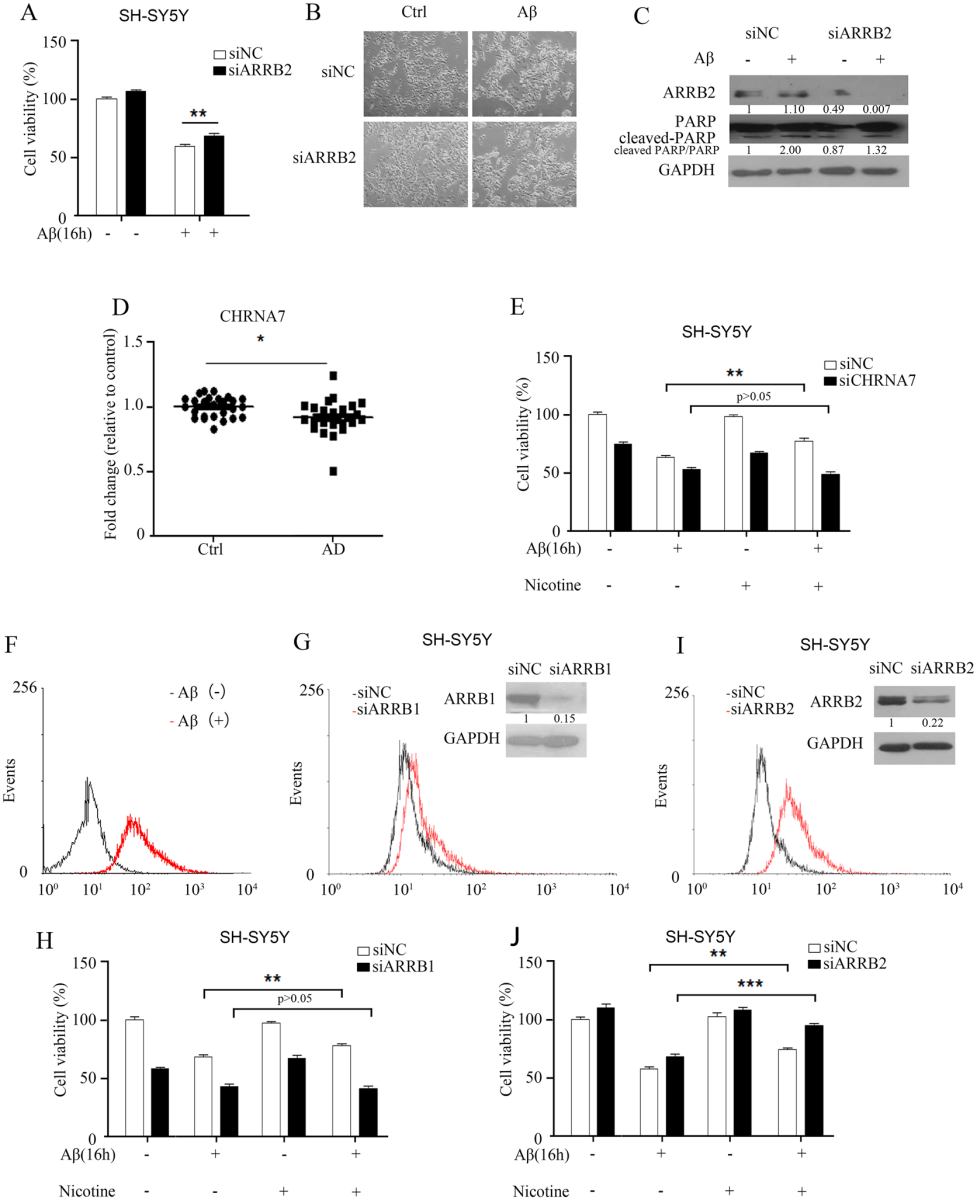
Knockdown of ARRB2 partially protects cells from Aβ_25–35_-induced apoptosis by facilitating α7nAChR expression at the plasma membrane. (**A**) After the depletion of ARRB2, Aβ_25-35_-induced cytotoxicity was evaluated with the MTT assay. **P < 0.01 compared with negative-control siRNA- and Aβ_25-35_-treated cells. (**B**) SH-SY5Y cells treated with Aβ_25-35_ after depletion of ARRB2; changes in cell morphology were observed with a microscope. (**C**) SH-SY5Y cells treated with Aβ_25-35_ after knockdown; the ARRB2 and apoptosis marker (PARP) was detected by western blot. (**D**) The mRNA level of *chrna7* was detected from peripheral blood of AD patients by RT-PCR. *P < 0.05 compared to *chrna7* mRNA of healthy controls. (**E**) After knockdown of *chrna7*, cells were treated with or without nicotine before Aβ_25-35_ treatment. **P < 0.01 compared to cells with negative-control siRNA and Aβ_25-35_ treatment and without nicotine pretreatment. (**F**) After Aβ_25-35_ treatment, the expression of α7nAChR in the cell membrane was detected by flow cytometry. (**G**) After *arrb1* knockdown, the expression of α7nAChR in the cell membrane was detected by flow cytometry. ARRB1 was detected by western blot. (**H**) After silencing of *arrb1*, cells pretreated with nicotine before exposure to Aβ_25-35_ were subjected to cell viability testing with the MTT assay. **P < 0.01 compared to cells with negative-control siRNA and Aβ_25-35_ treatment and without nicotine pretreatment. (**I**) After *arrb*2 knockdown, the expression of α7nAChR in the cell membrane was detected by flow cytometry. ARRB2 was detected by western blot. (**J**) After silencing of *arrb2*, cells pretreated with or without nicotine before exposure to Aβ_25-35_ were subjected to cell viability testing with the MTT assay. ***P < 0.001, **P < 0.01 compared to cells with negative-control siRNA and Aβ_25-35_ treatment and without nicotine pretreatment. The raw data of figure C/G/I is the figure 5C/5G/5I in supplemental data.

As ARRB2 was rapidly induced at 1 h and declined to basal levels following exposure to Aβ_25-35_ (Fig. 3A), we next eliminated ARRB2 gene expression by siRNA to examine the role of ARRB2 in Aβ_25-35_-mediated effects in SH-SY5Y cells. The results indicated that downregulation of ARRB2 slightly enhanced cell viability, as depletion of ARRB2 partially protected against Aβ_25-35_-induced cytotoxicity (Fig. 5A). Parallel experiments examining cell morphology changes again showed a decrease in apoptotic cells treated with Aβ_25-35_ after the depletion of ARRB2 (Fig. 5B). To confirm this effect of ARRB2 on Aβ_25-35_-induced apoptosis, we explored changes in the PARP cleavage by western blot analysis. As shown in Fig. 5C, knockdown of ARRB2 rescued Aβ_25-35_-mediated apoptosis, as evidenced by a reduction of cleaved PARP in cells treated with Aβ_25-35_, which is consistent with the observations shown in Fig. 5A and B.

Recognizing the role of β-arrestins in the internalization of G protein-coupled receptors (GPCRs), we decided to investigate the expression of the α7 nicotinic acetylcholine receptor (α7nAChR), a subtype of nicotinic acetylcholine receptors (nAChRs) that are widely distributed in the postsynaptic membrane of the neuron in the brain and offer protection against Aβ-induced toxicity^35, 36^. The mRNA of α7nAChR was markedly decreased in the peripheral blood of AD patients compared to healthy controls (Fig. 5D). This finding is consistent with previous reports indicating that the expression of α7nAChR decreases early in AD and correlates well with cognitive dysfunctions^37–39^.

Since nicotine is a α7nAChR agonist that can activate α7nAChR and offer protective effects from Aβ toxicity^36^, we first validated the effects of α7nAChR activity in the presence of Aβ_25-35_. As expected, compared to the control cells, knockdown of α7nAChR accelerated cell death, and cell viability as a result of nicotine treatment did not show significant recovery due to the loss of the α7nAChR (Fig. 5E). Moreover, Aβ_25-35_ induced cell death, and this cytotoxic effect was exacerbated by the depletion of α7nAChR (Fig. 5E), consistent with previous reports^36^.

Flow cytometry analysis revealed that Aβ treatment caused an increase in the expression ofα7nAChR at the cell membrane (Fig. 5F). We next asked whether the ARRB1/2-mediated effect was dependent on α7nAChR expression on the cell membrane. There was a small increase in the expression of α7nAChR in the cell membrane lacking ARRB1, as detected by flow cytometry (Fig. 5G). The cell viability assays revealed that silencing of *arrb1* exacerbated Aβ-induced toxicity, and nicotine pretreatment also did not improve the survival of cells exposed to Aβ when ARRB1 expression was low in cells (Fig. 5H). In contrast, down-regulation of ARRB2 significantly enhanced the abundance of α7nAChR (Fig. 5I), which in turn leading to improved survival, and enhancement of nicotine-mediated protection against Aβ (Fig. 5J). These findings suggest that ARRB2, but not ARRB1, played a critical role in the acceleration of Aβ toxicity, at least in part via the ability of ARRB2 to modulate α7nAChR expression on the cell membrane.

## Discussion

This study presents a novel role of ARRB1 and ARRB2 in Aβ_25-35_-induced neuronal cell death. Our results reveal that upregulation of ARRB1 and ARRB2 was an early event after Aβ_25-35_ exposure, associated with an induction of autophagy. Downregulation of *arrb1* led to the inactivation of autophagic flux and exacerbation of Aβ_25-35_-mediated cell death, whereas depletion of *arrb2*, to some extent, reversed the cytotoxicity of Aβ_25-35_. Our study further showed that, unlike ARRB1, which was critical for activation of autophagy, ARRB2 preferentially regulated α7nAch receptor expression on the membrane, which mediates the neuroprotective effect of nicotine. Knockdown of *arrb2* enhanced the expression of the α7nAch receptor at the plasma membrane, which in turn attenuated Aβ_25-35_ toxicity. β-arrestins have initially identified as desensitizers of canonical GPCR signaling, the β-arrestins are now recognized as regulators of G protein–independent signaling^40^. For example, transient receptor potential vanilloid 1 (TRPV1) is a nonselective cation channel activated by multiple stimuli, Por *et al*. have identified ARRB2 as a scaffolding protein that desensitizes TRPV1 receptor activity^41^. We also demonstrated that ARRB2 was able to regulate α7nAChR, because downregulation of ARRB2 facilitated the expression of α7nAChR on cell membrane, leading to the enhancement of nicotine on cell proliferation. Further investigation is required for elucidating the mechanism by which ARRB2 desensitizes α7nAChR activity.Aβ can suppress neuronal autophagy by impairing AMPK^23, 42^, leading to the reduced autophagic clearance of some aggregation-prone proteins and enhancing neuron cell death^24^. Basal levels of autophagy are essential for the removal and repurposing of damaged cytoplasmic contents and aggregated proteins, which is critical for cellular homeostasis. Therefore, many efforts have been made to ascertain whether the induction of autophagy would be beneficial for stimulating the clearance of Aβ, reducing the toxicity of Aβ in AD. For example, Justicidin A, an arylnaphthalide lignin, reduces Aβ_25-35_-mediated neuronal cell death by inhibiting hyperphosphorylation of tau and inducing autophagy in SH-SY5Y cells^43^. Cilostazol stimulates CK2/SIRT1 activation, resulting in upregulation of autophagy and a decrease in Aβ expression in neurons^44^. In contrast, Hung *et al*. demonstrated that extracellular Aβ induces a strong autophagic response in both SH-SY5Y cells and mice that overexpress LC3^45, 46^. These findings also indicate that α7nAChR binds with extracellular Aβ and the complex internalizes into the cytoplasm, subsequently inhibiting Aβ-induced neurotoxicity via autophagic degradation. We also found that Aβ_25-35_ treatment transiently activated autophagic flux in SH-SY5Y cells and eliminated autophagy after 4 h. Disruption of the autophagic process led to an increase in Aβ_25-35_-induced cell death, indicating that clearance of Aβ by the autophagosome provides neuroprotection. In addition, we demonstrated that incubation of cells with cytotoxic concentrations of soluble Aβ_25-35_ resulted in a rapid increase in the number of lysosomes and caused lysosomal damage after 4 h of incubation, consistent with observations previously reported^35^. We conclude that suppression of the autophagic process by longer treatments with Aβ may be a consequence of interrupted autophagosome maturation due to Aβ-induced damage of the lysosomal membrane.

Based on our cell model, using SH-SY5Y cells treated with Aβ_25-35_, we demonstrated that Aβ_25-35_ rapidly increased ARRB1 expression after short-term treatment, which contributed to the activation of autophagy, and the impairment of *arrb1* exacerbated Aβ-mediated cell death. In line with the reports that ARRB1 interacts with Beclin 1 and promotes activation of autophagy, deletion of *arrb1*, but not *arrb2*, aggravates neuronal injury in cerebral ischemia^30^. The findings from our study and others support a neuroprotective role of ARRB1 against neuronal injury in the regulation of autophagosome formation. We noted that mRNA levels of both *arrb1* and *arrb2*, particularly *arrb1*, were markedly reduced in blood samples of AD patients (Fig. 4A). However, β-arrestin levels have been correlated with Aβ toxicity in brains of AD patients and animal models^31, 32, 47^. These conflicting findings concerning the expression of β-arrestins in AD indicate that β-arrestin expression varied in tissue and blood samples. Further investigation is required to validate levels of the two proteins in a large sample, and to examine the correlation of β-arrestins with AD progression. AMPK is also activated by the exposure of cells to Aβ. Thornton *et al*. provided evidence that Aβ_1-42_ activates AMPK via the N-methyl-D-aspartate (NMDA) receptor, which in turn leads to the hyperphosphorylation of tau, a hallmark of AD^48^, although the authors did not show changes in autophagy upon the activation of AMPK in response to Aβ. It seems that the effect of Aβ on AMPK is context-dependent; under certain conditions, AMPK can be activated by Aβ, but in other contexts, AMPK is inhibited^49^. Research is still needed to clarify the role of AMPK in the autophagic process in AD. In addition to a regulatory effect of Aβ on ARRB1-induced autophagy, the expression of ARRB2 was enhanced after short treatment with Aβ. However, unlike ARRB1, genetic silencing of *arrb2* reduced the toxicity of Aβ in cell culture. In further experiments, knockdown of *arrb2* significantly increased the expression of the α7nAChR at the cell membrane, partially increased nicotine-mediated cytoprotective effects and rescued cells from Aβ_25-35_-induced cell death. Silencing of *arrb1* slightly facilitated α7nAChR expression, and the effect of *arrb2* on α7nAChR expression was more predominant in cells. To our knowledge, this is the first report demonstrating the role of the ARRB2/α7nAChR complex in Aβ_25-35_-mediated cytotoxicity. Huang *et al*. provided evidence that overexpression of LC3 in SH-SY5Y cells was associated with higher α7nAChR expression, which facilitated cell survival via internalization and autophagic degradation of extracellular^45, 46^. Given the role of β-arrestins in the recruitment of membrane receptors into intracellular compartments, it is reasonable to assume that the membrane α7nAChR could be recycled with the help of ARRB2, but further investigation is required to define this mechanism of action. Moreover, α7nAChR protein is reduced in the cortex and hippocampus in patients with AD^50^. Increased α7nAChR expression at the cell membrane could be beneficial for AD treatment^51^. Therefore, genetically or pharmacologically targeting ARRB2 with or without the use of nicotine or nicotinic ligands may have therapeutic potential in AD treatment^36^.

In this report, we observed the changes in ARRB1 and ARRB2 expression in response to Aβ_25-35_ and demonstrated the roles of ARRB1 and ARRB2 in Aβ_25-35_-mediated toxicity. We showed a neuroprotective role of ARRB1 in activating autophagy, which further confirms the importance of autophagy in neuroprotection. Moreover, our results indicate that therapies aimed at reducing ARRB2 may offer a promising approach for AD treatment because downregulation of ARRB2 enhances therapeutic effects mediated by α7nAChR.

## Materials and Methods

### Cell culture and treatments

The human neuroblastoma cell line SH-SY5Y was obtained from the Cell Resource Center, IBMS, CAMS/PUMC. Cells were cultured in RPMI-1640 medium (HyClone) supplemented with 10% fetal bovine serum (Gibco). The differentiated rat pheochromocytoma cell line PC12 was cultured in DMEM medium (HyClone) containing 5% fetal bovine serum (Gibco) and 10% horse serum (HyClone). Human embryonic kidney HEK293 cells and HEK293^APP^ cells (stably expressing APP) (a gift from Dr. Xiulian Sun, School of Medicine, Shandong University) were cultured in DMEM (HyClone) containing 10% fetal bovine serum (Gibco). The cells were maintained in 5% CO_2_ at 37 °C until reaching approximately 50–70% confluence, and then treated with Aβ_25-35_ as indicated. Control cells were cultured under normal conditions. Aβ_25-35_ was purchased from Sigma-Aldrich. Aβ_25-35_ was dissolved in double-distilled water to 5 mM·L^−1^ and incubated in 37 °C for 5 days before use. Control cells were exposed to equivalent volumes of double-distilled water.

### Cell viability assay

Cells were seeded in 96-well plates and were treated with Aβ_25-35_ for 16 h. Each test dose was performed in triplicate on each plate. The treated cells were then incubated with 10 μl MTT for 4 h at 37 °C, and the cell growth response to Aβ was detected by measuring the absorbance at 570 nm on a plate reader (Bio-Rad, USA). Three replicates were performed for each measurement.

### Transient transfection of plasmids and siRNAs

Cells were transfected with an ARRB1 expression plasmid using Lipofectamine 3000 (Invitrogen Life Technologies). After 24 h of transfection, cells were exposed to Aβ for an additional 16 h and subjected to further analysis. Control cells were transfected with the empty vector pcDNA3.1 under the same conditions. For the siRNA assay, ARRB1, ARRB2, LC3B, Beclin-1 or CHRNA7 siRNAs (Invitrogen Life Technologies) were transfected into cells for 48 h. Scrambled siRNA served as a control. After transfection, cells were exposed to chemicals as indicated and subjected to the cell viability assay or lysed for the western blot assay. At least three independent experiments were performed. The siRNA sequences were as follows: sense: 5′-GAGACGCCAGUAGAUACCAAUCUCA, anti-sense: 5′-UGAGAUUGGUAUCUACUGGCGUCUC for ARRB1; sense: 5′-GACCGACUGCUGAAGAAGUTT, anti-sense: 5′-ACUUCUUCAGCAGUCGGUCTT for ARRB2; sense: 5′-GCACCUUCGAACAAAGAGUTT, anti-sense: 5′-ACUCUUUGUUDGAAGGUGCTT for LC3B;sense: 5′-UGAAAUUUCAGACCCAUCUUAUUGG, antisense: 5′-CCAAUAAGAUGGGUCUGAAAUUUCA for Beclin-1;sense: 5′-GCUGGUCAAGAACUACAAUTT, anti-sense: 5′-AUUGUAGUUCUUGACCAGCTT for CHRNA7, the negative-controlsilencing RNA was used as a control. All of the experiments were performed intriplicate wells and repeated at least three times.

### Western blotting

After transfection and/or treatment with chemicals, cells were lysed for the western blot assay as described previously^52^. Bands were incubated with primary antibodies against PARP(SC-7150, Santa Cruz Biotechnology), glyceraldehyde-3-phosphate dehydrogenase (GAPDH) (SC-47724, Santa Cruz Biotechnology), β-actin(sc-47778, Santa Cruz Biotechnology), ARRB1 (ab32099, Abcam), ARRB2 (10171-1-AP, Proteintech), LC3B (NB100-2220), GRP78 (NBP1-06274), eIF2α(NB100-81896), and phosphor-eIF2α(NB110-56949) were purchased from Novus Biologicals, phosphor-p44/42MAPK(T202/Y204) (#4370S), phosphor-MEK(#9121S), phosphor-AKT(T308) (#9275S), ATG7 (#2631S), and Beclin-1 (#3738S) were purchased from Cell Signaling Technology.

### Immunofluorescence

Cells were seeded on coverslips in 24-well plates. After transfection and/or treatment, cells were fixed with a mixture of methanol and acetone (1:1) and permeabilized in phosphate-buffered saline (PBS) containing 3% BSA and 0.1% Triton X-100 for 20 min. After washing with PBS, cells were probed with primary antibodies overnight. The cells were incubated with peroxidase-conjugated secondary antibodies, and images were acquired using an LSM-700 confocal fluorescence microscope (Carl Zeiss, Germany).

### Flow cytometry

After transfection of ARRB1 or ARRB2 siRNAs for 48 h, cells were washed and re-suspended in PBS containing 5 mM EDTA and 0.2% BSA for 15 min. A monoclonal antibody against the nicotinic AChRα7 subunits (Abcam, UK) was added to the buffer, and the cells were incubated for 30 min on ice. The cells were then probed with FITC-conjugated secondary antibody for 30 min on ice in the dark, and analyzed on a FACScan flow cytometer (Becton Dickinson, USA).

### Real-time quantitative PCR

To evaluate changes in the mRNA levels of *arrb1* and *arrb2* in patients with AD, a total of 54 blood samples were collected from Chinese subjects (27 with AD and 27 healthy controls). The subjects were recruited from the Second Affiliated Hospital of Shandong University, Jinan. AD was clinically diagnosed according to the diagnosis guidelines spearheaded by the Alzheimer’s disease and the National Institute on Aging (NIA) of the National Institutes of Health (NIH). Informed consent was obtained from all participants, and all the experimental protocols involving human participants were approved by the medical ethics committee of Shandong University School of Medicine and Second hospital of Shandong University. And all the experimental protocols conducted in accordance with the ethica guidelines of the Declaration of Helsinki of the World Medical Association. Total RNA was isolated from venous blood mixed with ethylenediamine tetra-acetic acid (EDTA) utilizing the RiboPure™-Blood Kit (ThermoFisher) in accordance with the manufacturer’s instructions. Complementary DNA was synthesized by reverse transcription using ReverTra Ace qPCR RT Kit (Toyobo, Japan). Quantitative PCR analysis of cDNA was performed using SYBR Green (Toyobo) on a real-time PCR system (Eppendorf International, Germany). The mRNA levels of the desired genes were normalized to glyceraldehyde 3-phosphate dehydrogenase (GAPDH). The following primer pairs were used: *arrb1* primers: 5′-AAAGGGACCCGAGTGTTCAAG-3′, 5′-CGTCACATAGACTCTCCGCT-3′; *arrb2* primers: 5′-TCCATGCTCCGTCACACTG-3′, 5′-ACAGAAGGCTCGAATCTCAAAG-3′; *gapdh* primers: 5′-GGAGCGAGATCCCTCCAAAAT-3′, 5′-GGCTGTTGTCATACTTCTCATGG-3′.

### Statistical analysis

All experiments were performed at least three independent times in triplicate. Results are expressed as mean ± standard deviation (SD). The two-tailed Student’s *t*-test was performed to assess differences between the experimental and control groups. ARRB1, ARRB2, and cell viability under conditions of depletion and treatment were measured and presented as mean ± standard error of the mean (SEM), and values were compared by two-way analysis of variance (ANOVA). P < 0.05 was considered statistically significant; P ≤ 0.001 was considered highly significant. The fluorescence intensity of images were analyzed by Image J software.

## Electronic supplementary material

Supplementary Information
