# Supplementary material for: Arrestins contribute to amyloid beta-induced cell death via modulation of autophagy and the α7nAch receptor in SH-SY5Y cells
Source: Sci Rep. 2017 Jun 13;7:3446. doi: 10.1038/s41598-017-01798-x (PMC5469748; doi:10.1038/s41598-017-01798-x)
Supplement: Supplementary file 1 — Supplementary Information [file 41598_2017_1798_MOESM1_ESM.pdf]

Arrestins contribute to amyloid beta-induced cell death via modulation of autophagy and the α7nAch receptor in SH-SY5Y cells

Yi-qing Liu[1], Meng-qi Jia[2], Zhao-hong Xie[1], Xiao-fei Liu[2], Hui-Yang[1], Xiao-lei Zheng[1], Hui-qing Yuan[2]*, Jian-zhong Bi[1]*

[1]Department of Neural Medicine/Key Laboratory of Translational Medicine on Neurological Degenerative Disease, Second Hospital of Shandong University, Jinan 250033, China

[2]Department of Biochemistry and Molecular Biology, School of Medicine, Shandong University, Jinan 250012, China

Correspondence to: JianZhong Bi, Department of Neural Medicine, Second Hospital of Shandong University, Jinan 250033, China; Tel: +86 531 85875006, Fax: +86 531 88962544, Email: bjz@sdu.edu.cn; Hui-Qing Yuan, Department of Biochemistry and Molecular Biology, School of Medicine, Shandong University, Jinan 250012, China. Tel.: +86 531 88382346; E-mail: lyuanhq@sdu.edu.cn.

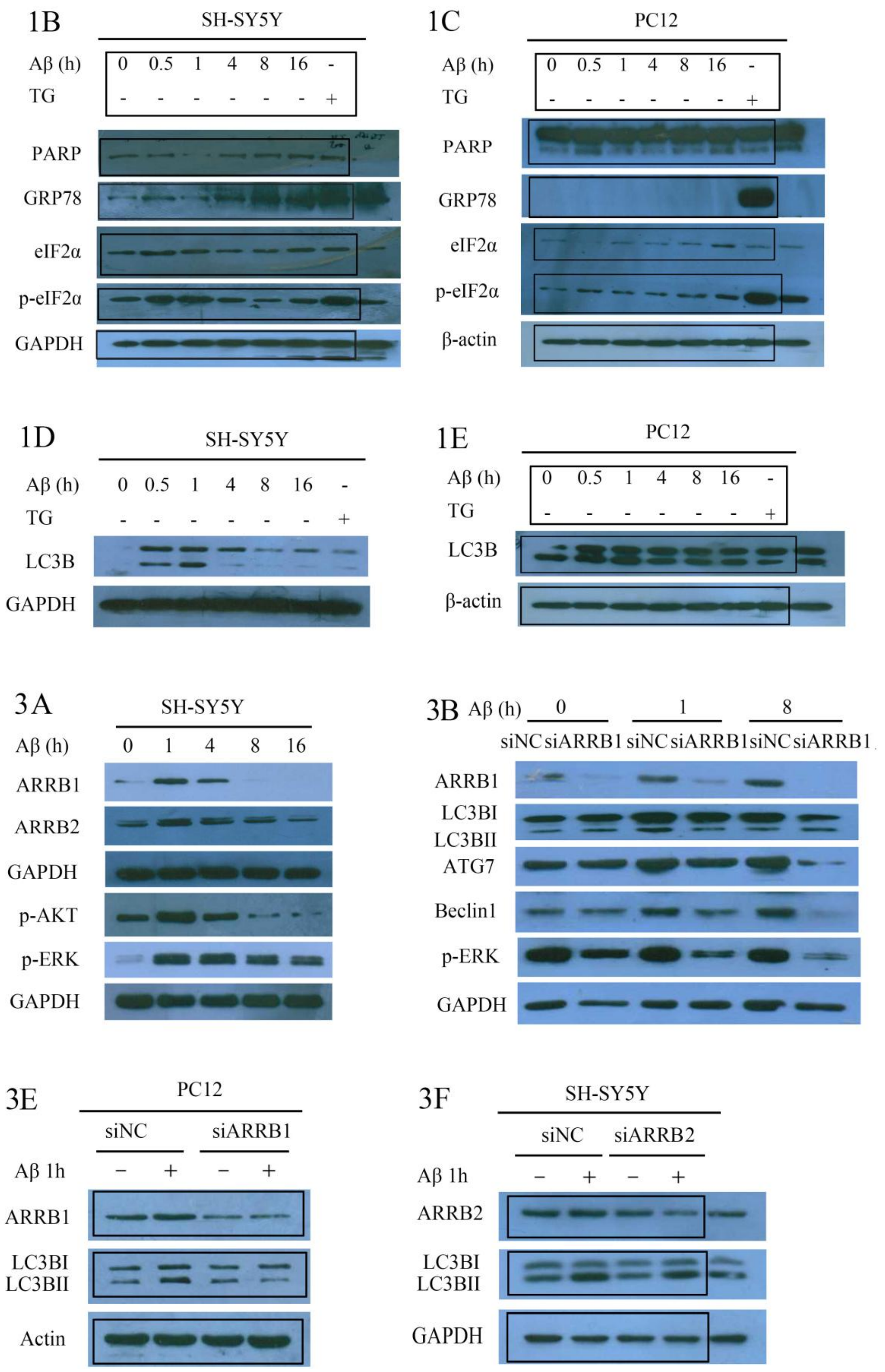
1B
SH-SY5Y
Aβ (h) 0 0.5 1 4 8 16 -
TG - - - - - - +
PARP
GRP78
eIF2α
p-eIF2α
GAPDH
1C
PC12
Aβ (h) 0 0.5 1 4 8 16 -
TG - - - - - - +
PARP
GRP78
eIF2α
p-eIF2α
β-actin
1D
SH-SY5Y
Aβ (h) 0 0.5 1 4 8 16 -
TG - - - - - - +
LC3B
GAPDH
1E
PC12
Aβ (h) 0 0.5 1 4 8 16 -
TG - - - - - - +
LC3B
β-actin
3A
SH-SY5Y
Aβ (h) 0 1 4 8 16
ARRB1
ARRB2
GAPDH
p-AKT
p-ERK
GAPDH
3B Aβ (h) 0 1 8
siNC siARRB1 siNC siARRB1 siNC siARRB1
ARRB1
LC3BI
LC3BII
ATG7
Beclin1
p-ERK
GAPDH
3E
PC12
siNC siARRB1
Aβ 1h − + − +
ARRB1
LC3BI
LC3BII
Actin
3F
SH-SY5Y
siNC siARRB2
Aβ 1h − + − +
ARRB2
LC3BI
LC3BII
GAPDH

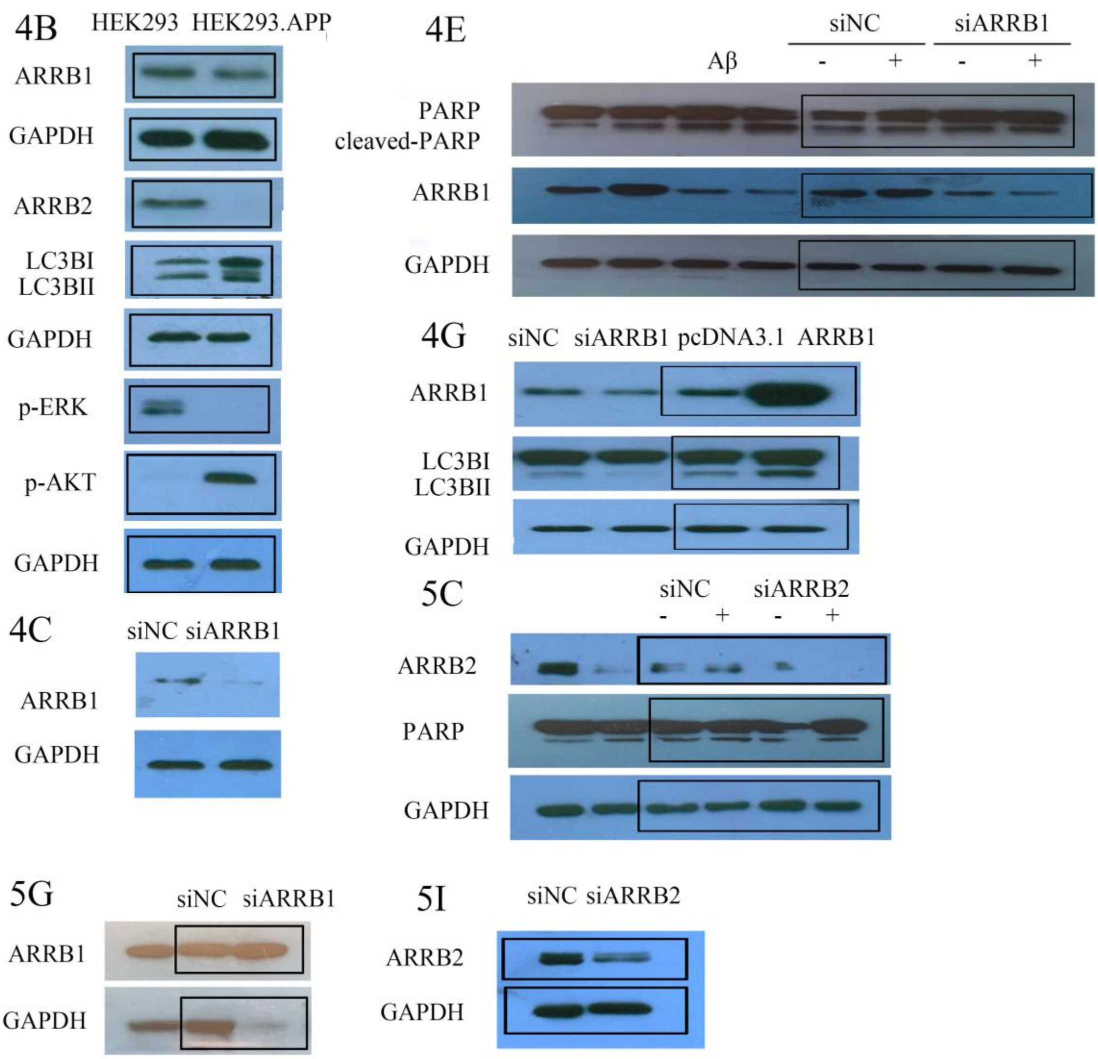
4B
HEK293 HEK293.APP
ARRB1
GAPDH
ARRB2
LC3BI
LC3BII
GAPDH
p-ERK
p-AKT
GAPDH
4C
siNC siARRB1
ARRB1
GAPDH
4E
siNC
siARRB1
Aβ
-
+
-
+
PARP
cleaved-PARP
ARRB1
GAPDH
4G
siNC siARRB1 pcDNA3.1 ARRB1
ARRB1
LC3BI
LC3BII
GAPDH
5C
siNC
siARRB2
-
+
-
+
ARRB2
PARP
GAPDH
5G
siNC siARRB1
ARRB1
GAPDH
5I
siNC siARRB2
ARRB2
GAPDH
